# LC-MS/MS Determination of Quorum Sensing Molecules in Plasma from Burn Patients with Septic Shock Sustained by Acinetobacter Baumannii

**DOI:** 10.3390/antibiotics14050517

**Published:** 2025-05-16

**Authors:** Nicolò Carpenito, Marta Leporati, Alberto Sciarrillo, Anna Pensa, Roberto Gambino, Giovanni Musso, Alberto Mella, Luigi Biancone, Daniela Risso, Filippo Mariano, Domenico Cosseddu

**Affiliations:** 1 Department of Public Health and Pediatric Sciences, University of Turin, 10126 Turin, Italy; carpenito.nic@gmail.com; 2 Laboratory of Analytical Chemistry and Kidney Stones, Analysis Laboratory, Mauriziano Hospital, 10128 Turin, Italy; mleporati@mauriziano.it (M.L.); dcosseddu@mauriziano.it (D.C.); 3 Plastic Surgery and Burn Center, Department of General and Specialized Surgery, City of Health and Science, CTO Hospital, 10126 Turin, Italy; albertosciarrillo@gmail.com (A.S.); anna.pensa@unito.it (A.P.); drisso@cittadellasalute.to.it (D.R.); 4 Department of Medical Sciences, City of Health and Science, University of Turin, 10126 Turin, Italy; roberto.gambino@unito.it; 5 MECAU Department, San Luigi Gonzaga Hospital, Orbassano, 10043 Turin, Italy; giovanni.musso@yahoo.it; 6 Nephrology, Dialysis and Transplantation U, Department of Medical Sciences, City of Health and Science, University of Turin, 10126 Turin, Italy; alberto.mella@unito.it (A.M.); luigi.biancone@unito.it (L.B.)

**Keywords:** quorum sensing molecules, LC-MS/MS, *Acinetobacter baumanni*, N-acyl-homoserine lactones, septic shock, burns

## Abstract

**Background/Objectives**: Quorum Sensing (QS) refers to the communication mechanism in bacterial cells, which occurs through the production and detection of small signaling molecules to coordinate activities and monitor population size. In Gram-negative bacteria, QS is typically mediated by N-acyl-homoserine lactones (HSLs) and 2-alkyl-4(1H)-quinolone metabolites (AQ). The present study aims to develop and validate an LC-MS/MS method for detecting QS molecules and apply it to the analysis of plasma samples from burn patients with septic shock caused by *Acinetobacter baumannii*. **Methods**: The LC-MS/MS method was developed and fully validated for the quantitative, simultaneous determination of five HSLs and four AQ molecules, ultimately derived from the plasma of three patients with septic shock, with samples collected over three consecutive days. **Results**: The developed method proved to be both specific and selective, demonstrating a good fit and linearity over the entire range of interest. Trueness and accuracy were satisfactory. The method showed excellent intra-assay precision (CV% was lower than 15%) and limits of quantification (LOQ) ranging from 0.02 to 0.79 ng/mL. In the patients’ samples, the concentration of 3-OH-C12-HSL peaked at 1.5 ng/mL on the first day, and C7-PQS, C9-PQS, HHQ, and HQNO ranged from 0.5 to 1.5 ng/mL, peaking at 5 ng/mL in one patient on the third day. **Conclusions**: A method for the simultaneous determination of nine QS molecules by LC-MS/MS was developed and validated. When applied, it showed good performance for the analysis of plasma samples and could be a useful tool for an improvement in the diagnosis, prognosis, or treatment monitoring of infections in burn patients caused by *Acinetobacter baumannii*.

## 1. Introduction

Antibiotic-resistant bacteria represent a serious global challenge, largely driven by the widespread and improper use of antibiotics. The discovery of the quorum sensing (QS) mechanism, which allows microorganisms to perceive and respond to their environment, has provided new insights into the treatment of infectious diseases [1]. QS refers to the communication mechanism in bacterial cells, enabling them to produce and sense small signaling molecules to coordinate activities and monitor population size [2]. Identifying the signaling molecules involved in the QS system of infectious microorganisms may enhance the effectiveness of infection treatments [3].

In Gram-negative bacteria, QS is typically mediated by N-acyl-homoserine lactone (HSL) signaling molecules [2]. These molecules are characterized by an γ-lactone ring, which is N-acylated at the α position, and an acyl chain. The length of the acyl chain determines signal specificity for bacteria, typically ranging from 4 to 16 carbon atoms. Additional specificity is provided by the presence of an oxo or hydroxy group attached to the third carbon atom of the chain [4,5]. *Acinetobacter baumannii* primarily produces HSLs with acyl side chains ranging from 10 to 16 carbon atoms. Although some studies have reported HSLs with C6 and C8 acyl side chains, the predominant molecule is 3-OH-C12-HSL [6,7,8,9]. In the human opportunistic pathogen *Pseudomonas aeruginosa*, QS is also mediated by 2-alkyl-4(1H)-quinolone (AQ) metabolites, which exhibit a range of biological activities during infection. These compounds can be categorized into 2-alkyl-4(1H)-hydroxyquinolones (AHQs), 2-alkyl-4-hydroxyquinolone N-oxides (AQNOs), and 2-alkyl-3-hydroxy- 4(1H)-quinolones (PQS). Variations in alkyl chain length and saturation result in over 50 different AQ congeners [3].

One promising strategy for diagnosing bacterial infections involves using QS molecules as biomarkers. However, the full potential of this approach and the biological roles of these molecules remain poorly understood. In this context, advanced techniques such as immunochemical assays, biosensors, and liquid chromatography–tandem mass spectrometry (LC-MS/MS) have been developed to detect and quantify QS molecules in both bacterial cultures and clinical samples [10].

Immunochemical assays can detect QS molecules through the specific binding between antigens and antibodies. However, these traditional detection methods are directed to a single QS molecule [11] or an entire QS molecule family [12]. In contrast, biosensors have gained increasing interest in biomolecule detection due to their unique physicochemical characteristics. The bioreceptor could specifically recognize and interact with the targets, while the transducers could convert the identifying information into detectable signals such as optical, electrochemical, magnetic, or acoustic signals. They have the potential to significantly enhance the detection performance of QS molecules, but practical applications are still hindered by several challenges [13]. As an alternative, LC-MS/MS methods are particularly notable for their ability to accurately quantify HSLs and AQ compounds, allowing for the simultaneous determination of a wide range of QS molecules, even from different families, including enantiomeric forms [14,15].

Critically ill polytrauma patients, including those with burn injuries, constitute a group at high risk of infections. In burn patients, Gram-negative bacteria are among the major pathogens responsible for sepsis and septic shock due to their high virulence and antibiotic resistance [16]. Among these bacteria, *Acinetobacter* and *Pseudomonas* strains can significantly impact patient survival by inducing profound metabolic changes, sustained by their growth, virulence, and biofilm production—processes closely linked to the production and release of QS molecules, such as HSL signaling molecules and AQ metabolites [17]. Therefore, measuring QS molecules could be important in this particular patient population, leading to better management or treatment of these patients.

To address this issue, we focused on the *A. baumannii* QS molecules most frequently identified in previous studies [2,3,4,5,6,7,8,9]. Despite the fact that *A. baumannii* primarily produces HSLs, AQ molecules were also included because of the availability of a powerful technology that allows us to expand the range of target molecules. Analyzing a broad spectrum of QS molecules can provide a more comprehensive overview of the microbial environment and its dynamics, allowing for a better understanding of the infection.

**Figure 1:**
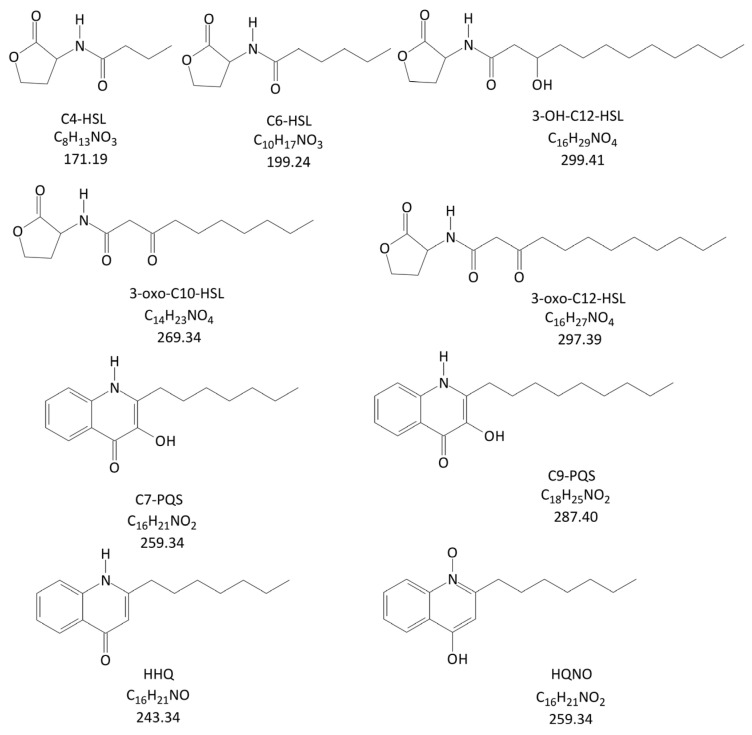
Chemical structures of all tested analytes.

Therefore, we developed and validated an LC-MS/MS method for the simultaneous determination of nine QS molecules (five HSLs and four AQs; see Figure 1) and applied it to the analysis of plasma samples from severe burn patients with septic shock caused by *A. baumannii* infection.

## 2. Results

### 2.1. Method Development

**Figure 2:**
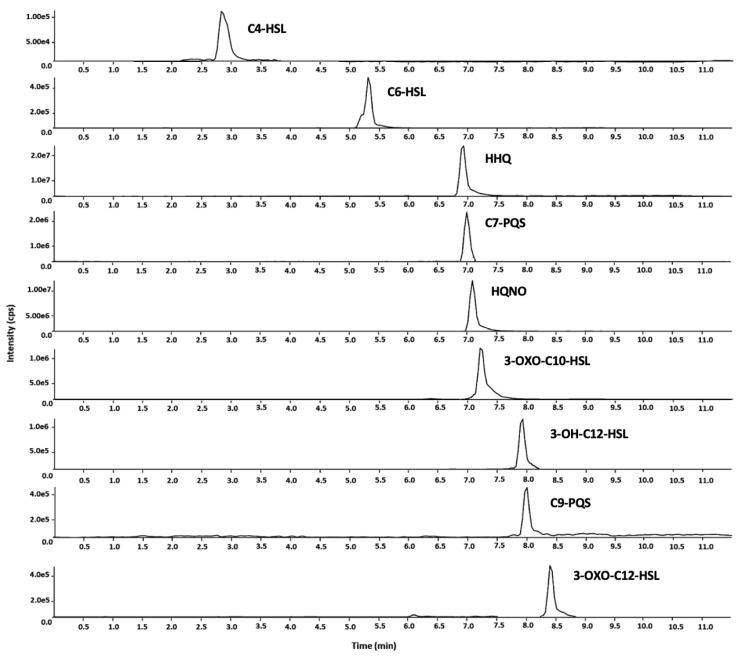
SRM chromatograms (quantifier fragment) for all the analytes of a mixed standard solution at 100 ng/mL. Legend: C4-HLS = N-butanoyl-L-homoserine lactone; C6-HLS = N-butanoyl-L-homoserine lactone; HHQ = 2-heptyl-4-quinolone; C7-PQS = 2-Heptyl-3-hydroxy-4(1H)-quinolone; HQNO = N-oxo-2-heptyl-4-Hydroxyquinoline; 3-OXO-C10-HSL = N-(3-oxodecanoyl)-L-homoserine lac-tone; 3-OH-C12 = N-(3- hydroxydodeca-noyl)-DL-homoserine lactone; C9-PQS = 2-nonyl-3-hydroxy-4(1H)-quinolone; 3-OXO-C12-HSL = N-(3-oxododecanoyl)-L-homoserine lactone.

The optimized LC-MS/MS method enabled the simultaneous determination of nine QS molecules in human plasma. The entire chromatographic run, including the time required for column re-equilibration before the subsequent injection, was completed in 11.5 min. Retention times ranged from 2.89 min (C4-HSL) to 9.41 min (3-oxo-C12-HSL). Figure 2 presents the selected reaction monitoring (SRM) chromatograms recorded from a standard mixture at a concentration of 100 ng/mL.

### 2.2. Validation Results

The validation results are summarized in Table 1. The SRM chromatographic profiles obtained from QS-free matrix samples collected from six untreated subjects did not show any significant signal (signal-to-noise ratio (S/N) < 3) at the retention times corresponding to the studied compounds. This indicates that the method is selective and free from interfering substances in biological matrices.

The linearity of the matrix-matched calibration model was evaluated by analyzing two replicate QS-free matrix samples spiked with working solutions at six final concentrations. Quantitative data obtained from area counts were corrected using the respective internal standard (IS) signal areas, yielding R² values ranging from 0.9911 to 0.9995. The limit of quantification (LOQ) values ranged from 0.02 to 0.79 ng/mL, while recovery rates varied between 46% and 87%, except for 3-oxo-C10-HSL and 3-oxo-C12-HSL, which showed a recovery of only 8%. This low recovery was presumably due to the presence of esterase enzymes in the plasma matrix, leading to the partial hydrolysis of QS molecules, which are esters [18]. However, this issue was mitigated by using calibration curves obtained from plasma matrices.

Following the main guidelines on bioanalytical method validation [19,20,21,22], the freeze–thaw stability of stock standard solutions always fell within the acceptable limits (85–115%) for all the analytes. Precision and accuracy data demonstrated satisfactory repeatability, with coefficient of variation (CV%) values below 15% for all spiked analytes at low, medium, and high concentrations. The only exceptions were two inter-run values (25% and 17% for HQNO at low and medium levels, respectively). Accuracy was also satisfactory, with percent bias (bias%) ranging from −15% to +15%, except for two cases (23% for HQNO and 16% for C6-HSL, both at low levels).

Regarding carry-over, the background chromatographic profiles of the main SRM transitions for each analyte, monitored during the analysis of QS-free matrix samples injected after highly spiked samples, did not show any significant signal (i.e., S/N < 3) at the expected retention times of the tested analytes (Table 2).

### 2.3. Results for Real Samples

**Figure 3:**
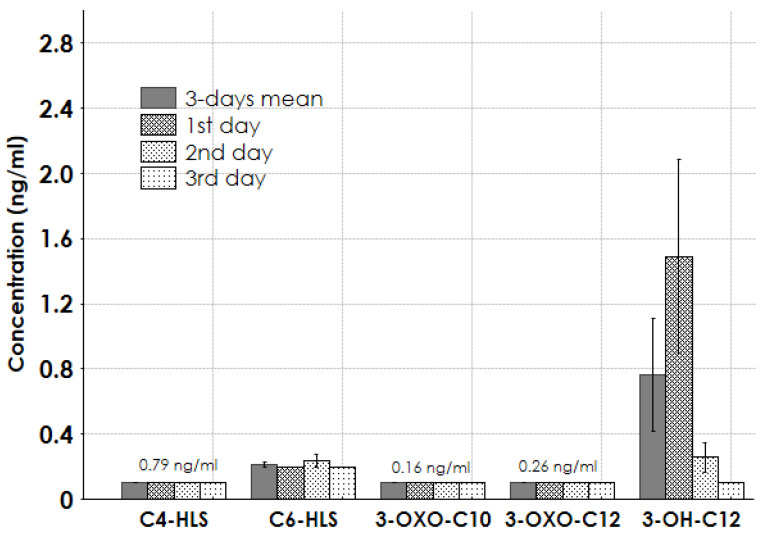
Concentrations of HSL metabolite group in patients’ samples. Legend: C4-HLS = N-butanoyl-L-homoserine lactone; C6-HLS = N-butanoyl-L-homoserine lactone; 3-OXO-C10 = N-(3-oxodecanoyl)-L-homoserine lactone; 3-OXO-C12 = N-(3-oxododecanoyl)-L-homoserine lactone; 3-OH-C12 = N-(3- hydroxydodecanoyl)-DL-homoserine lactone.

Among the various HSL molecules, only C6-HSL and 3-OH-C12-HSL were present at significant concentrations in the patient samples. Specifically, 3-OH-C12-HSL had a mean concentration of 0.8 ng/mL over three days, peaking at 1.5 ng/mL on the first day (Figure 3). No detectable concentrations of C4-HSL, 3-oxo-C10-HSL, or 3-oxo-C12-HSL were found.

**Figure 4:**
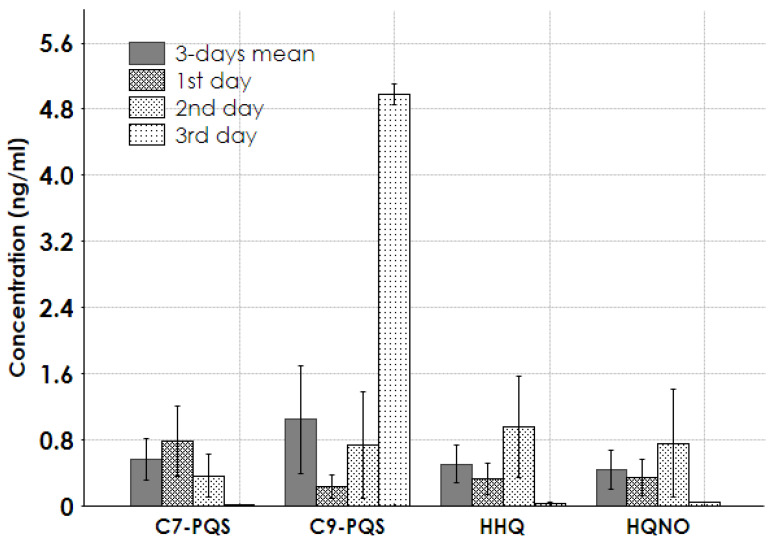
Concentrations of AQ metabolite group in patients’ samples. C7-PQS = 2-Heptyl-3-hydroxy-4(1H)-quinolone; C9-PQS = 2-nonyl-3-hydroxy-4(1H)-quinolone; HHQ = 2-heptyl-4-quinolone; HQNO = N-oxo-2-heptyl-4-Hydroxyquinoline.

Figure 4 illustrates the concentrations of QS molecules in the AQ metabolite group. The concentrations of C7-PQS, C9-PQS, HHQ, and HQNO ranged from 0.5 to 1.5 ng/mL, with a peak of 5 ng/mL observed in one patient on the third day.

**Figure 5:**
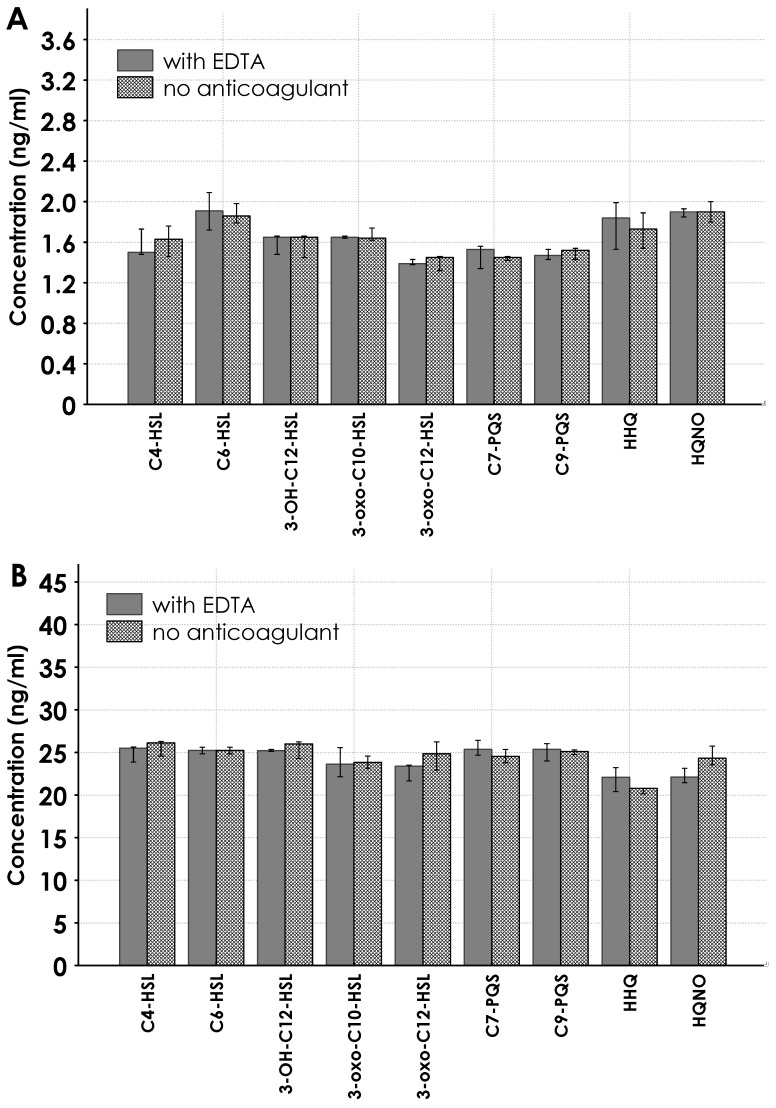
Controls of serum samples obtained from tubes with EDTA and without anticoagulant or separator. Controls were performed with concentrations of 1.6 ng/mL (panel **A**) and 25 ng/mL (panel **B**) of the nine QS molecules (five replicates for each group). Mann–Whitney U test *p* > 0.05.

No significant difference was found between the sample controls obtained from tubes with EDTA and those without an anticoagulant or separator. The controls were performed for the nine QS molecules at 1.6 and 25 ng/m concentrations (see Figure 5A,B).

## 3. Discussion

In the present study, an LC-MS/MS method for the simultaneous determination of nine QS molecules (five HSLs and four AQs) was developed and validated following the main guidelines on bioanalytical method validation. The method demonstrated good performance and was successfully applied to the analysis of plasma samples from severe burn patients with septic shock caused by *A. baumannii*.

Numerous studies in the literature report analytical methods for detecting QS molecules in biological samples. The primary techniques include electrochemical sensors, immunochemical-based approaches, and, more recently, LC-MS/MS. The working ranges of electrochemical sensors are generally on the micromolar scale, which is suitable for bacterial culture samples but insufficient for clinical samples. Immunochemical-based techniques offer the advantage of assessing the biological and clinical significance of QS molecules with minimal sample preparation. However, only LC-MS/MS enables the accurate quantification of QS molecules [14,23]. Moreover, LC-MS/MS provides high specificity and allows for the simultaneous detection of multiple analytes in a single run. Specifically, our method facilitates the detection of both HSL and AQ molecules in a single chromatographic analysis. Only a few LC-MS/MS methods have been reported in the literature. Brewe et al. developed an LC-MS/MS method for the detection of only AQ molecules [3], while the method proposed by Chen et al. targets only HSL molecules [13]. Dal Bello et al. [18] validated their LC-MS/MS method in multiple-reaction monitoring mode for just three analytes, two HSLs, and one AQ. Nevertheless, the use of this approach on clinical samples remains limited, and further applications are needed to gain a deeper understanding of QS processes. The choice of electrospray ionization (ESI) in positive mode was based on its higher sensitivity for both HSL and AQ analytes [14]. Although ordinary production was observed for each family (*m*/*z* 102 for HSL, *m*/*z* 159 for AQ, and *m*/*z* 175 for PQS), optimal results were obtained using a specific type of production as the quantifier MRM transition for each analyte (see Table 2).

Regarding chromatographic separation, previous studies have reported poor peak shapes, particularly for PQS molecules [3,14]. This chromatographic behavior is attributed to the known metal-chelating properties of PQS molecules [24]. The use of an acidic mobile phase and the addition of competing soluble metal chelators compatible with MS analysis improved PQS peak shapes, but this effect was not stable over time. On the other hand, adding EDTA, a non-volatile chelating agent, could lead to ionization suppression and increase the risk of MS system contamination [14]. A more effective solution was found using the highly volatile bidentate chelator 2-picolinic acid. The 2-picolinic acid addition to the aqueous mobile phase significantly enhanced PQS peak shapes and also promoted efficient positive ESI in the MS [23].

The choice of liquid–liquid extraction was made based on a comparison with literature data [20]. Our goal was to use the LC-MS/MS technique to evaluate a panel of the most common QS molecules in cases of *A. baumannii* infections, despite the lack of unambiguous indications in the literature [3,6,7,8,9]. Thus, we selected the QS molecules most frequently detected in previous studies.

The primary QS molecules in *A. baumannii* are typically *N*-acyl homoserine lactones (HSLs) [6,7,8,9]. These molecules mediate bacterial communication and regulate virulence factors. As expected, the QS molecule 3-OH-C12-HSL was detected in plasma samples at significant concentrations (Figure 3). Moreover, we also found high levels of the AQ Pseudomonas Quinolone Signal (PQS) in plasma samples collected on the third day of a septic shock episode in a patient with positive hemoculture for *A. baumannii*, *Enterococcus faecium*, and *Candida parapsilosis*. Notably, AQ PQS is commonly associated with the presence of *P. Aeruginosa* [3]. PQS and its metabolites have been documented in bacterial cultures and play a role in regulating virulence factors, such as biofilm formation, immune modulation, and the production of elastase and pyocyanin [25]. In humans, PQS and structurally related molecules have been detected in sputum and tissue samples from cystic fibrosis patients infected with *P. aeruginosa* [26]. Therefore, we cannot exclude the possibility that the presence of an AQ-like PQS molecule in a septic shock episode with a positive hemoculture for *Acinetobacter* could indicate co-infection with multiple pathogens, including *Pseudomonas* species.

In burn patients, *A. baumannii* and *P. aeruginosa* are often nosocomial pathogens with overlapping sites of infection. Microorganisms produce antimicrobial substances regulated by QS to suppress competing microorganisms in the same ecological niche. Structurally similar quinolone molecules may be produced or mutually utilized by both *Acinetobacter* and *Pseudomonas* species [27,28,29]. Specifically, pyocyanin, a virulence factor produced by *P. aeruginosa* to eliminate competing pathogens, generates reactive oxygen species in *A. baumannii* cells. This oxidative stress response significantly increases the production of catalase and superoxide dismutase (SOD) enzymes, which, in *A. baumannii*, are regulated by the QS system [30].

The present paper reported preliminary data on QS plasma determinations for the specific ICU population of burn patients with *A. baumannii*-related septic shock.

We are aware that our study has some limitations. First, a simple validation protocol was applied and considered sufficient for the aim of our work. The influence of matrix effects was compensated for by using a matrix-matched external standard calibration prepared in plasma with a labeled internal standard for each molecular family [22]. The potential for interference was studied during the selectivity evaluation [19,20,21], as reported below in the paragraph “Section 4.5”. Secondly, regarding the real sample determinations, the sample size number of studied subjects was limited, and we used a very specific population. However, this second point could also be considered a strength of our study, as it is limited to a population with well-defined clinical characteristics that meet the accuracy criteria required for modern precision medicine [31]. In terms of clinical utility, hemoculture, procalcitonin, C-reactive protein, and lactate levels are always crucial for diagnosing septic shock in burn patients. However, our data suggested that LC-MS/MS is a competitive technique in terms of feasibility and cost-effectiveness compared with other complicated techniques. Today, this technique is available for routine analysis in analytical laboratories. Given that the analysis is based on QS standards, the analysis excludes other signal molecules that are not in the category of QS molecules. More information could be added through QS determinations to improve patient outcomes, guide treatment decisions, or predict prognosis in burn patients with sepsis.

Therefore, these results, far from being conclusive, should be considered a starting point for further studies.

## 4. Materials and Methods

### 4.1. Chemicals

N-butanoyl-L-homoserine lactone (C4-HSL), N-hexanoyl-L-homoserine lactone-d_3_ (C6-HSL-d3), 2-nonyl-3-hydroxy-4(1H)-quinolone (C9-PQS), 2-nonyl-3-hydroxy-4- Quinolone-d4 (C9-PQS-d4), and N-oxo-2-heptyl-4-Hydroxyquinoline (HQNO) were purchased from Cayman Chemical (Ann Arbor, MI, USA).

N-hexanoyl-L-homoserine lactone (C6-HSL), N-(3-oxodecanoyl)-L-homoserine lactone (3-oxo-C10-HSL), N-(3-oxododecanoyl)-L-homoserine lactone (3-oxo-C12-HSL), 2-Heptyl-3-hydroxy-4(1H)-quinolone (C7-PQS), 2-heptyl-4-quinolone (HHQ), N-(3-hydroxydodecanoyl)-DL-homoserine lactone (3-OH-C12-HSL), 2-picolinic acid, ethyl acetate, formic acid for LC-MS, and acetonitrile for LC-MS were purchased from Merck (Darmstadt, Germany). All reagents were of analytical grade.

Ultrapure water (conductivity < 0.05 μS/cm; ASTM parameter Type I) was obtained by using a 1720 Deionizer Device (G. Maina, Pecetto Torinese, Torino, Italy).

### 4.2. Standard and Working Solutions

Stock standard solutions of all analytes and ISs (C6-HSL-d3 for the HSL family and C9-PQS-d4 for the AQ family) were prepared in methanol, at a concentration of 1000 µg/mL. Working standard solutions were obtained by proper dilutions. Stock and working standard solutions were stored at −20 °C in the dark.

### 4.3. Sample Preparation

Blood samples were collected into tubes with anticoagulant EDTA. After centrifugation at 3500 rpm for 5 min, plasma was collected and transferred to a new tube. Then, 300 µL of the sample was transferred into an Eppendorf tube, and 750 µL of ethyl acetate containing ISs at 100 ng/mL was added. The Eppendorf tube was vortexed and centrifuged at 10,000 rpm for 5 min. The supernatant was transferred to a collection plate, and another 750 µL of ethyl acetate containing ISs was added to the residue. The Eppendorf tube was again vortexed and centrifuged at 10,000 rpm for 5 min, and the supernatant was mixed with the previous one. All supernatant was evaporated under a nitrogen stream at 40 °C. The residue was collected with 100 µL of the initial mobile phase, and 10 µL was injected into the LC-MS system.

### 4.4. Instrumental Analysis

The chromatographic separation was performed with a Nexera liquid chromatograph (Sciex, Concord, ON, Canada), including a vacuum degasser, a binary pump, an autosampler, and a column thermostat. The liquid chromatograph was equipped with an Acquity UPLC BEH C18 Column, 130 Å, 1.7 µm, 2.1 mm × 100 mm, and an Acquity UPLC BEH C18 VanGuard Pre-column, 130 Å, 1.7 µm, 2.1 mm × 5 mm (Waters S.p.A., Sesto San Giovanni (MI), Italy). The chromatographic run was carried out using gradient conditions. Mobile phase A was 0.1% HCOOH in ultrapure water with picolinic acid at a concentration of 2 mM, and mobile phase B was 0.1% HCOOH in acetonitrile with picolinic acid at a concentration of 2 mM. The mobile phase was eluted under the following conditions: (A/B; *v*/*v*): initial 78:22 ratio for 1 min, linear gradient to 5:95 in 7.5 min and to 0:100 for 1 min. The total run time was 9.5 min, plus 2 min of re-equilibration time. The injection volume was 10 µL, and the flow rate was 0.2 mL/min. The LC was interfaced to a Sciex 6500 Qtrap mass spectrometer (Sciex, ON, Canada), operating in the positive ion mode for electrospray ionization (ESI). The other MS parameters were set as follows: curtain gas, 35 psi; collision gas, “medium”; ion spray voltage, 5500 V; probe temperature, 550 °C; ion source gas-1, 40 psi; ion source gas-2, 40 psi. Ion acquisition was carried out at unit mass resolution in the selected reaction monitoring (SRM) mode. The SRM ion transitions, together with the relative values for declustering potential, entrance potential, collision energy, and cell exit potential, are reported in Table 1.

### 4.5. Method Validation

A simple validation protocol was applied: selectivity, calibration curve, LOQ, accuracy, precision, recovery, and carry-over were determined [19,20,21]. A matrix free of QS molecules was used for the validation experiments following the analytical protocol described below.

The freeze–thaw stability of stock standard solutions was evaluated. This was determined under the storage conditions used during the analysis of the study samples (−20 °C for six months) [22].

For selectivity evaluation, six matrices free of QS molecules from different healthy individuals were analyzed. The occurrence of possible interferences from endogenous substances was tested by monitoring the SRM chromatogram characteristics for each investigated compound at the expected retention time interval. The S/N was measured, and a value lower than 3 was considered satisfactory to verify the method’s specificity.

The linear calibration model was checked by analyzing a matrix free of QS molecules spiked at six concentration levels (0.78, 1.56, 3.12, 6.25, 12.5, and 25.0 ng/mL) for all the analytes. The calibration was completed by internal standardization, and each concentration level was measured in duplicate. In addition, a blank sample (QS-free matrix sample) was processed but not considered among the calibration curve parameters. The linear calibration parameters were evaluated using the least-squares regression method, and LOQ levels were calculated with the S/N calculation approach [19].

Precision was also evaluated in inter-run conditions: three QC replicates for each concentration level were analyzed in three different analytical runs on three different days by the same analyst and with the same equipment.

Recovery was evaluated by relating the responses of analytes in the extracted samples to those solubilized in the injection solvent.

Intra-run accuracy (bias%) and precision (CV%) were evaluated on the QS-free matrix sample spiked at three concentration levels (1.56, 6.25, and 25.0 ng/mL for all analytes; n = 3) analyzed in the same batch and across different days. Precision was also evaluated in inter-run conditions. Intra-run precision was considered satisfactory when CV% values were below 15% [19,20,21]. Satisfactory accuracy was achieved when the experimentally determined average concentration was within ±15% of the expected value [19,20,21].

Carry-over was evaluated by injecting an alternate sequence with the highest point on the calibration curve and a QS-free matrix sample. To ensure the absence of any carry-over effect, S/N ratios had to be lower than 3 at the specified analyte retention times in the QS-free matrix sample chromatograms. A measured S/N value lower than 3 was considered satisfactory to verify the method specificity. To evaluate possible interference from EDTA [32], serum samples obtained from tubes without anticoagulant or separator gel were tested, and no significant differences were observed (Figure 5).

### 4.6. Patients and Real Sampling Protocol

Three serial critically ill burn patients, admitted to the Burn Unit at CTO Hospital with severe septic shock sustained by infection and hemoculture positive for multi-drug-resistant *A. baumanni*, were studied. Out of the 3 patients, the youngest patient with 85% burn extent had a fatal outcome resulting from multiorgan failure after 8 months of hospitalization.

The clinical baseline characteristics of the studied patients are shown in Table 3.

All samples were drawn directly from the systemic arterial blood of patients in the early morning for 3 consecutive days. Plasma and effluent samples were immediately stored at −25 °C and were analyzed within three months.

The study was conducted according to the Helsinki Declaration and approved by the Ethics Committee of the City of Health and Science (protocol code n. 66875 on 14 June 2022).

Informed consent to the proposed treatments and consent to retrospectively review the medical notes and analyze the collected data were obtained from the patients or substitute decision-makers.

### 4.7. Statistical Analysis

The software program Statistica (Statistica 10.2, StaSoft Inc., Tulsa, OK, USA) was used for descriptive statistics and graphs. All values are expressed as median (interquartile range).

The Mann–Whitney U test was applied when appropriate.

## 5. Conclusions

LC-MS/MS proved to be a competitive technique in terms of feasibility, cost-effectiveness, precision, and accuracy to detect QS molecules in the plasma of burn patients with septic shock sustained by Gram-negative bacteria. By carrying out LC-MS/MS in patients with hemoculture positive for *A. baumannii*, the QS molecule 3-OH-C12-HSL, commonly associated with this strain, was detected at significant concentrations. Besides 3-OH-C12-HSL, high levels of PQS, a molecule commonly associated with the presence of *P. Aeruginosa*, were also found in plasma samples from a patient with positive hemoculture for *A. baumannii*, *Enterococcus faecium*, and *Candida parapsilosis.* Given the critical role of these signaling molecules in the communication and virulence regulation of different bacterial competing microorganisms, their simultaneous quantification via LC-MS/MS could improve the accuracy and effectiveness of infection treatment strategies.

## Figures and Tables

**Table 1:** R^2^, LOQ, intra-day and inter-day precision (CV%), and intra-day accuracy (bias%) for all the analytes tested.

Analyte	R^2^	LOQ (ng/mL)	Concentration (ng/mL)	Within-Run (n = 3)		Inter-Run (n = 3)
				Precision (CV%)	Accuracy (Bias%)	Precision (CV%)
C4-HSL	0.9984	0.79	1.56	8.2	+1.3	9.6
			6.25	6.3	−1.0	2.0
			25.0	6.0	+1.3	0.1
C6-HSL	0.9995	0.15	1.56	11	+16	11
			6.25	1.0	−2.2	6.8
			25.0	0.6	−0.1	12
HHQ	0.9957	0.15	1.56	9.6	+3.2	15
			6.25	9.1	+4.2	9.4
			25.0	10	−2.4	10
HQNO	0.9911	0.02	1.56	13	+23	25
			6.25	10	−15	17
			25.0	17	−7	15
3-oxo-C10-HSL	0.9954	0.16	1.56	7.5	+1.7	8.2
			6.25	3.9	−6.2	4.1
			25.0	5.7	−0.9	5.8
C7-PQS	0.9992	0.05	1.56	9.3	−4.3	8.8
			6.25	3.1	−14	11
			25.0	3.5	+1.9	7.5
3-oxo-C12-HSL	0.9959	0.26	1.56	13	−4.3	11
			6.25	2.0	−11	6.4
			25.0	11	−6.6	9.6
C9-PQS	0.9932	0.03	1.56	3.0	−0.1	24
			6.25	8.0	+0.3	8
			25.0	15	−0.1	9
3-OH-C12-HSL	0.9992	0.62	1.56	9.3	+5.8	0.2
			6.25	10	+11	13
			25.0	6.6	+4.6	1.6

Legend: C4-HLS = N-butanoyl-L-homoserine lactone; C6-HLS = N-butanoyl-L-homoserine lactone; HHQ = 2-heptyl-4-quinolone; HQNO = N-oxo-2-heptyl-4-Hydroxyquinoline; 3-OXO-C10-HSL = N-(3-oxodecanoyl)-L-homoserine lac-tone; 3-OXO-C12-HSL = N-(3-oxododecanoyl)-L-homoserine lactone; C9-PQS = 2-nonyl-3-hydroxy-4(1H)-quinolone; 3-OH-C12 = N-(3- hydroxydodeca-noyl)-DL-homoserine lactone.

**Table 2:** SRM transitions and corresponding potentials for the detection of target compounds and internal standards.

Analyte	t_R_ (Min)	Precursor Ion	DP (V)	EP (V)	Product Ion	CE (V)	CXP (V)
C4-HSL	2.89	172.1	30	15	102.2	14	12
					71.1	19	12
C6-HSL	5.37	200.3	30	13	102.0	15	15
					99.0	15	15
HHQ	6.95	244.3	35	11	172.0	47	20
					159.2	43	10
C7-PQS	7.00	260.3	25	13	175.2	39	20
					147.1	50	20
HQNO	7.15	260.3	30	10	159.0	41	15
					186.0	50	22
3-oxo-C10-HSL	7.24	270.3	25	13	169.0	18	11
					102.1	18	10
3-OH-C12-HSL	7.97	300.2	25	6	282.2	13	20
					102.1	17	14
C9-PQS	8.02	288.4	40	14	175.3	41	14
					147.0	52	20
3-oxo-C12-HSL	8.41	298.4	31	13	197.2	20	18
					102.1	17	20
C6-HSL-d3	5.28	203.3	25	14	102.2	15	10
C9-PQS-d4	7.99	292.2	35	5	179.3	45	12

DP: declustering potential; EP: entrance potential; CE: collision energy; CXP: cell exit potential. C4-HLS = N-butanoyl-L-homoserine lactone; C6-HLS = N-butanoyl-L-homoserine lactone; HHQ = 2-heptyl-4-quinolone; C7-PQS = 2-Heptyl-3-hydroxy-4(1H)-quinolone; HQNO = N-oxo-2-heptyl-4-Hydroxyquinoline; 3-OXO-C10-HSL= N-(3-oxodecanoyl)-L-homoserine lactone; 3-OH-C12 = N-(3- hydroxydodeca-noyl)-DL-homoserine lactone; C9-PQS = 2-nonyl-3-hydroxy-4(1H)-quinolone; 3-OXO-C12-HSL = N-(3-oxododecanoyl)-L-homoserine lactone; C6-HSL-d3 = N-hexanoyl-L-homoserine lactone-d3; C9-PQS-d4 = 2-nonyl-3-hydroxy-4- Quinolone-d4.

**Table 3:** Clinical characteristics of septic shock patients.

	Age/Sex	Diagnosis	Day Samples	Hemoculture	Exitus
1.	89/F	Burns 15%	1-2-3	yes/*Acinetobacter baumanni/Candida parapsilosis/Enterococcus faecium*	No
2.	43/M	Burns 45%	1-2	yes/*Acinetobacter baumanni/Candida albicans*	No
3.	19/F	Burns 85%	1-2	yes/*Acinetobacter baumanni*	Yes

## Data Availability

The datasets used and analyzed during the current study are available from the corresponding author upon reasonable request.

## Abbreviations

The following abbreviations are used in this manuscript:

AQ	2-alkyl-4(1H)-quinolone metabolites
HSL	N-acyl-homoserine lactone
QS	Quorum Sensing
